# The Diagnostic Role of ^18^F-Choline, ^18^F-Fluciclovine and ^18^F-PSMA PET/CT in the Detection of Prostate Cancer With Biochemical Recurrence: A Meta-Analysis

**DOI:** 10.3389/fonc.2021.684629

**Published:** 2021-06-17

**Authors:** Rang Wang, Guohua Shen, Mingxing Huang, Rong Tian

**Affiliations:** Department of Nuclear Medicine, West China Hospital, Sichuan University, Chengdu, China

**Keywords:** PET/CT, ^18^F, choline, fluciclovine, prostate-specific membrane antigen, prostate cancer

## Abstract

**Background:**

Diagnosing the biochemical recurrence (BCR) of prostate cancer (PCa) is a clinical challenge, and early detection of BCR can help patients receive optimal treatment. We conducted a meta-analysis to define the diagnostic accuracy of PET/CT using ^18^F-labeled choline, fluciclovine, and prostate-specific membrane antigen (PSMA) in patients with BCR.

**Methods:**

Multiple databases were searched until March 30, 2021. We included studies investigating the diagnostic accuracy of ^18^F-choline, ^18^F-fluciclovine, and ^18^F-PSMA PET/CT in patients with BCR. The pooled sensitivity, specificity, and detection rate of ^18^F-labeled tracers were calculated with a random-effects model.

**Results:**

A total of 46 studies met the included criteria; 17, 16, and 13 studies focused on ^18^F-choline, fluciclovine, and PSMA, respectively. The pooled sensitivities of ^18^F-choline and ^18^F-fluciclovine were 0.93 (95% CI, 0.85–0.98) and 0.80 (95% CI, 0.65–0.897), and the specificities were 0.91 (95% CI, 0.73–0.97) and 0.66 (95% CI, 0.50–0.79), respectively. The pooled detection rates of ^18^F-labeled choline, fluciclovine and PSMA were 66, 74, and 83%, respectively. Moreover, the detection rates of ^18^F-labeled choline, fluciclovine, and PSMA were 35, 23, and 58% for a PSA level less than 0.5 ng/ml; 41, 46, and 75% for a PSA level of 0.5–0.99 ng/ml; 62, 57, and 86% for a PSA level of 1.0–1.99 ng/ml; 80, 92, and 94% for a PSA level more than 2.0 ng/ml.

**Conclusion:**

These three ^18^F-labeled tracers are promising for detecting BCR in prostate cancer patients, with ^18^F-choline showing superior diagnostic accuracy. In addition, the much higher detection rates of ^18^F-PSMA showed its superiority over other tracers, particularly in low PSA levels.

**Systematic Review Registration:**

PROSPERO, identifier CRD42020212531.

## Introduction

Prostate cancer (PCa) is one of the most common malignancy in men worldwide and is also the fifth major cause of cancer-related death in men. It is estimated that over 300,000 PCa-related deaths occur in 2018 (1). In addition to its high morbidity and mortality, the recurrence and metastasis of prostate cancer are also troublesome in clinical practice (2, 3).

It is challenging to detect initial recurrence and metastasis after prior treatment because of few obvious characteristics on early recurrent or metastatic lesions. PCa recurrence is usually considered when observing a rise in the serum prostate-specific antigen (PSA) level. This is regarded as biochemical recurrence (BCR) of PCa, and the definition of BCR is a serum PSA level over a threshold of 0.2 ng/ml twice after radical prostatectomy (RP) or an absolute increase in PSA level of 2 ng/ml over the lowest posttreatment PSA level after radiation therapy (RT) (4, 5).

The key issue for patients with BCR is the early and correct identification of recurrent or metastatic disease, which is essential for further devising treatment strategies since treatment varies based on the presence of local recurrence, regional lymph node and distant viscera or bone metastasis (6). Conventional imaging modalities consisting of CT, bone scan, and MRI have been used for patients with advanced PCa, but their roles in detecting minimal or occult lesions are limited (7, 8). These conventional imaging modalities also have low sensitivity and specificity in detecting patients with BCR, especially those with a low PSA level. According to the 2020 American Society of Clinical Oncology (ASCO) guidelines, next-generation imaging (NGI) such as PET/CT, PET/MRI, and whole-body MRI is recommended for use in patients with rising PSA after prior treatment when conventional imaging findings are negative (9). Radioactive tracers such as choline and fluciclovine have been used for prostate cancer staging, restaging, and treatment response evaluation. Meanwhile, prostate-specific membrane antigen (PSMA), a new radiopharmaceutical that binds to prostate cancer-specific target, has demonstrated outstanding detection rate for recurrent or metastatic lesions among patients with BCR.

Choline is an essential element of phospholipids in the cellular wall, and the increased uptake of choline means increased metabolism of the cell membrane components of malignant tumors (10). ^11^C-choline was approved by the Food and Drug Administration (FDA) in 2012, but its short half-life limits its widespread use in PET/CT centers without onsite cyclotrons. Later, ^18^F-labeled choline was developed, and its longer half-life has solidified ^18^F-choline PET/CT as a significant imaging modality in patients with suspected PCa recurrence (11, 12). ^18^F-Fluciclovine (anti-1-amino-3-^18^F-fluorocyclobutane-1-carboxylic acid, ^18^F-FACBC), as a synthetic amino acid that is upregulated in PCa, is an option for molecular imaging in patients with BCR, which was approved by the FDA in 2016 (13, 14). The main advantage of ^18^F-fluciclovine is its low urinary excretion, which allows for better detection and localization of PCa recurrence in patients with rising PSA level (15). PSMA is a type 2 transmembrane protein that is more highly expressed in the prostate cancer cell membrane than in normal tissues (16–18). Therefore, PSMA has become a promising target for imaging prostate cancer (19). ^68^Ga-PSMA PET/CT has been proven to improve the detection of metastatic disease and the monitoring of treatment effects in patients with PCa (20). Most recently, ^68^Ga-PSMA-11, as the first PSMA PET agent, has been approved by the FDA. ^18^F-labeled PSMA has also begun to be used in clinical practice, and its long half-life and high resolution in PET/CT images have further increased the detection rate of PSMA-targeted imaging in subtle or occult metastases (21, 22). Moreover, ^18^F-PSMA-1007 PET/CT can differentiate local recurrence from physical uptake in the urinary bladder or ureter due to non-urinary clearance (23, 24).

Some previous studies have compared the diagnostic roles of ^11^C-choline, ^18^F-fluciclovine, and ^68^Ga-PSMA PET/CT in patients with BCR, showing that ^68^Ga-PSMA PET/CT has a superior detection rate (25). Even so, there have been some clinical challenges existing in ^68^Ga-PSMA PET/CT due to certain shortcomings including its short half-life, non-ideal energies and the limited availability of ^68^Ga. Compared with ^68^Ga and ^11^C, ^18^F, as a longer half-life nuclide, has many advantages such centralized production in a cyclotron facility and more favorable positron energies for imaging, thereby motivating the development of ^18^F-labeled analogs. Currently, growing clinical experience has revealed the high diagnostic accuracy of some ^18^F-labeled tracers in PCa patients with BCR. However, the effectiveness of ^18^F-labeled choline, fluciclovine, and PSMA remains unclear because of limited number of studies. Herein, we aimed to perform a meta-analysis to review and compare the diagnostic value of ^18^F-labeled choline, fluciclovine, and PSMA PET/CT imaging for detecting BCR in patients with PCa, in order to provide better creditability for clinical practice.

## Methods

The Preferred Reporting Items for Systematic Reviews and Meta-analyses (PRISMA) guidelines was used for our study (26). Our review has registered on the international prospective register of systematic reviews (PROSPERO) (CRD 42020198861).

### Search Strategy

A literature research was conducted with scientific databases, including PubMed, EMBASE, and Web of Science, until March 30, 2021. A search algorithm was developed based on a combination of keywords (“choline” OR “fluciclovine” OR “FACBC” OR “PSMA” OR “DCFPyL” OR “DCFBC” OR “1007”) AND (“prostate cancer” OR “prostate neoplasm”) AND (“biochemical recurrence” OR “biochemical failure”) AND (“PET/CT” OR “positron emission tomography/computed tomography”) AND (“^18^F” OR “fluorine”).

Two authors independently screened and evaluated these studies. The reference lists of all relevant studies were further checked to find more suitable studies. A third author was responsible for disagreement and solved the controversy between two authors through discussion.

### Selection of Studies

Studies using ^18^F-labeled tracers such as ^18^F-choline, ^18^F-fluciclovine, and ^18^F-PSMA were evaluated. Studies were included according to the following criteria: (a) sample size >10; (b) patients who had evidence of BCR underwent PET/CT; (c) studies evaluating the diagnostic accuracy of ^18^F-labeled tracers in prostate cancer patients with BCR; (d) histological results, imaging, or clinical follow-up as a reference standard. Studies on other tracers were not included. Abstracts, reviews, and case reports were also not included. If the studies included duplicate patients, we reviewed and included the study with the largest sample or the most recent study performed. The included studies were limited to those published in English.

### Quality Assessment

The quality of included studies was critically assessed by two independent authors according to the Quality Assessment of Diagnostic Accuracy Studies-2 (QUADAS-2) tool. This tool comprises four domains (patient selection, index test, reference standard, and flow and timing), and each domain was used to assess the risk of bias. Next, applicability was also considered according to patient selection, the index test, and the reference standard.

### Data Extraction

Two authors collected various parameters and outcomes from each eligible study as follows: author, country, publication year, study design, number of patients, age, pre-PET PSA level, reference criteria, scanner model, ligands, and injection dose and the detection rate as well as true positive (TP), false positive (FP), false negative (FN), and true negative (TN) PET/CT with different tracers in patients with BCR. All discrepancies were resolved by consensus and ultimately based on the decision of the third author.

### Statistical Analysis

For studies reporting the diagnostic performance of ^18^F-PSMA, ^18^F-choline, and ^18^F-fluciclovine PET/CT in patients with BCR, 2 × 2 table was used to calculate TP, FP, TN, and FN. The pooled sensitivity and specificity were calculated by a random-effects model. We developed a hierarchical summary receiver operating curve and calculated the area under the curve. We presented forest plots with 95% confidence intervals (CIs) for the sensitivity and specificity of each study. In addition, the detection rates of PET tracers were extracted and pooled using a random-effects model. If possible, subgroup analysis was considered based on different PSA serum values.

Heterogeneity within studies was evaluated using Cochran’s Q test and the I² statistic (27). An I² value greater than 50% was indicative of substantial heterogeneity. The funnel plot test and Egger’s test were used to assess the publication bias. All statistical analyses were performed using Stata 15.0 and RevMan 5.3. P-value <0.05 was considered to be statistically significant (28).

## Results

**Figure 1:**
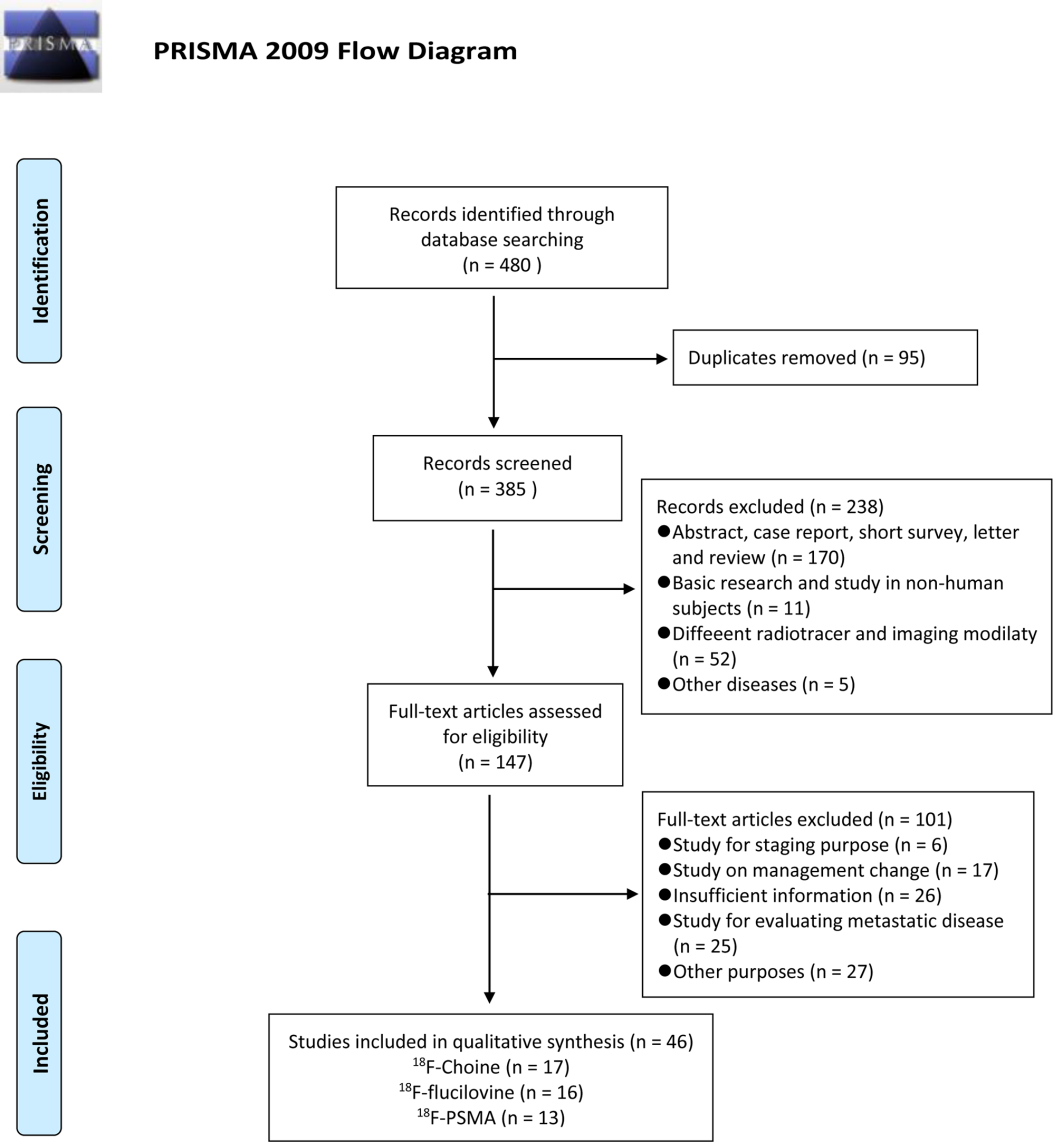
Flow of study search.

The flow chart demonstrates an overview of the search and selection process (**Figure 1**). The initial search yielded 480 studies, of which 95 were duplicates. Subsequently, after reviewing the titles and abstracts, we excluded 238 studies for the following reasons: 170 studies were case reports, reviews, and academic meeting abstracts, 11 were basic studies, five applications in other diseases, and 52 studies used different radiotracers and imaging modalities. Of the remaining studies, 75 studies were not relevant to our aims, and most of them investigated the impact of novel PET/CT tracers in treatment management for patients with PCa or focused on evaluating metastatic disease. In addition, 26 studies did not provide sufficient information and were excluded. Thus, only 46 studies were finally included. Of these, 17 studies focused on the role of ^18^F-choline PET/CT in prostate cancer patients with BCR (29–45). The numbers of included studies regarding ^18^F-fluciclovine and ^18^F-PSMA PET/CT were 16 and 13, respectively (46–74). **Tables 1**–**3** outline the characteristics of each eligible study.

**Table 1:** Study characteristics of ^18^F-choline PET/CT.

Author	Publication Year	Country	Design	Patients	Mean age/Range	PSA (ng/mL)	Scancer Modality	Ligand	Mean dose	Reference standard
Pelosi	2008	Italy	R	56	67.9 ± 7	4.59 ± 7.87	Discovery ST unit, GE	^18^F-choline	185–259 MBq	Multiple
Kwee	2012	US	P	50	69.0 ± 8.9	3.2 (0.2–18.2)	Philips Gemini TF-64	^18^F-fluorocholine	2.6 MBq/kg	Multiple
Panebianco	2012	Italy	P	84	56–72	1.63	Discovery ST, GE	^18^F-choline	185-259 MBq	Multiple
Schillaci	2012	Italy	P	49	70. 9 ± 7	4.13 ± 4.56	Discovery ST, GE	^18^F-choline	370 MBq	Biopsy
Detti	2013	Italy	R	170	——	3.5 ± 8.8	PET/CT Philips TOF	^18^F-fluorocholine	3.7 MBq/kg	Follow-up
Marzola	2013	Italy	R	233	69.4 ± 6.5	7.4 ± 13.6	Discovery STE	^18^F-fluorocholine	3 MBq/kg	
Piccardo	2014	Italy	P	21	77.2 ± 5.1	5.8 ± 3.4	Discovery ST, GE	^18^F-choline	3MBq/kg	Multiple
Morigi	2015	Australia	P	38	68(54–81)	1.72 ± 2.54	Philips Ingenuity TF 64	^18^F-fluorocholine	3.5 MBq/kg	Biopsy
Rodado-Marina	2015	Spain	R	233	68 ± 7.1	5.3 ± 8.7	GE and Siemens equipments	^18^F-fluorocholine	4 MBq/kg	Multiple
Cimitan	2015	Italy	R	1,000	69.68 ± 7.67	3.30	GE Discovery LS; Siemens Biograph 16 HT or Biograph mCT; GE Discovery ST8,	^18^F-choline	3.0–3.5 MBq/kg	Multiple
Simone	2015	Italy	P	146	68	0.6 (0.43–0.76)	Siemens Biograph Hi-Rez 16;	^18^F-choline	4 MBq/kg	Multiple
Emmett	2018	Australia	P	91	64 (59–69)	0.42 (0.29–0.93)	——	^18^F-choline	3.6 MBq/kg	Multiple
Giovacchini	2019	Italy	R	192	73.2 ± 6.6	9.53 ± 16.70	GE Discovery 710	^18^F-fluorocholine	3.7 MBq/kg	Multiple
Witkowska-Patena	2019	Poland	P	40	68.6 ± 6.5	0.75 ± 0.6	GE Discovery 710	^18^F-fluorocholine	248 ± 35 MBq	Multiple
Sánchez	2020	Spain	P	108	69 ± 6.7	4.9 ± 5.2	Siemens Biograph mCT	^18^F-choline	370 MBq	Multiple
Zattoni	2020	Italy	R	2,798	72 (66.29–77.0)	2.0 (0.1–3.0)	——	^18^F-choline	2.5-3.7 MBq/kg	
de Leiris	2020	France	R	115	73.2 (56–89)	9.4 (7.1–18.4)	GE Discovery 690	^18^F-choline	3.7 MBq/kg	Multiple

A multiple reference standards including biopsy, other imaging modalities and follow-up.

**Table 2:** Study characteristics of ^18^F-fluciclovine PET/CT.

Author	Publication Year	Country	Design	Patients	Mean age/Range	PSA (ng/mL)	Scancer Modality	Ligand	Mean dose	Reference standard
Schuster	2011	US	P	50	68.3 ± 8.1	6.62 ± 7.63	Discovery DLS, GE	^18^F-fluciclovine	199.8–484.7 MBq	Multiple
Kairemo	2014	Finland	R	26	68.1 ± 5.8	7.9 ± 14.6	Siemens Biograph	^18^F-fluciclovine	328 ± 56.8 MBq	Multiple
Nanni	2016	Italy	P	89	69	6.99	Discovery STE, GE	^18^F-fluciclovine	370 MBq	Follow-up
Odewole	2016	USA	R	53	67.57± 8.03	7.2 ± 8.3	GE Discovery DLS or 690	^18^F-fluciclovine	358 ± 52.9MBq	Follow-up
Bach-Gansmo	2017	Norway, Italy, UK	R	143	67	5.43	——	^18^F-fluciclovine	310 MBq	Follow-up
Miller	2017	USA	R	110	67.4 ± 7.37	5.87 ± 7.65	GE Discovery DLS or 690	^18^F-fluciclovine	370 MBq	Multiple
Akin-Akintayo	2018	USA	P	24	70.8 ± 5.7	8.5 ± 6.1	GE Discovery 690	^18^F-fluciclovine	370 ± 13 MBq	Biopsy
Jambor	2018	Finland	P	32	65 (49–76)	12 (4.1–35)	GE Discovery 690	^18^F-fluciclovine	369 ± 10 MBq	Biopsy
Andriole	2019	US	P	213	66.4 ± 7.75	4.24 ± 10.22	——	^18^F-fluciclovine	370 ± 20% MBq	Multiple
Calais	2019	US	P	50	68 (64–74)	0·48 (0·38–0·83)	Siemens Biograph64 and GE Discovery	^18^F-fluciclovine	381 MBq	Multiple
England	2019	US	R	28	67.1 (53–77)	0.44(0.1–1)	Siemens Biograph	^18^F-fluciclovine	370 MBq	Follow-up
Savir-Baruch	2019	US	R	152	68.73 ± 7.92	2.06 (0.006–120)	Philips Ingenuity TF PET/CT	^18^F-fluciclovine	9.97 ± 1.18mci	——
Teyateeti	2020	US	R	94	65.7 (42.5–80.3)		GE Discovery 710, MI and Siemens Biograph 64	^18^F-fluciclovine	370 MBq	Multiple
Garza	2021	US	R	78	68.7 (48–87)	0.72(<0.05–1.99)	——	^18^F-fluciclovine	——	Imaging
Michael	2021	US	R	103	69.79 ± 7.88	5.77 ± 9.98	Siemens Biograph	^18^F-fluciclovine	10 mci	Imaging
Nakamoto	2021	US	R	165	71.1 ± 8.8	3.1 (1.0–9.6)	GE Discovery 600, 690, or MI	^18^F-fluciclovine	389 ± 59 MBq	Multiple

A multiple reference standards including biopsy, other imaging modalities and follow-up.

**Table 3:** Study characteristics of ^18^F-PSMA PET/CT.

Author	Publication Year	Country	Design	Patients	Mean age/year	PSA (ng/ml)	Scancer Modality	Tracer	Mean dose	Reference standard
Rahbar	2018	Germany	R	100	68.75 ± 7.6	3.3 6 ± 6.11	Siemens Healthcare	^18^F-PSMA-1007	4 MBq/kg	——
Eiber	2018	Germany	R	261	72 (49–88)	0.961 (0.01–400.0)	Siemens Biograph mCT	^18^F‐rhPSMA‐7	333 ± 44 MBq	——
Giesel	2018	Germany	R	251	70 (48–86)	1.2 (0.2–228)	Siemens Biograph mCT	^18^F-PSMA-1007	301 ± 46 MBq	——
Rousseau	2018	Canada	P	130	69.1 ± 6.5	5.20 ± 6.50	GE Discovery PET/CT 600 or 690	^18^F-DCFPyL	369.2 ± 47.2 MBq	——
Wondergem	2019	Netherlands	R	248	71 (67–75)		Philips Ingenuity TF, Siemens Biograph 16	^18^F-DCFPyL	311 MBq	——
Mena	2019	USA	P	90	66 (50–81)	2.5 ± 5.9	GE Discovery MI	^18^F-DCFPyL	299.9 ± 15.5 MBq	——
Song	2019	USA	P	72	71.5 ± 7.2	3.0 (0.23-698.4)	GE Discovery MI	^18^F-DCFPyL	338.8 ± 25.3 MBq	——
Witkowska-Patena	2019	Poland	P	40	68.6 ± 6.5	0.75 ± 0.6	GE Discovery 710,	^18^F-PSMA-1007	295.5 ± 14.1 MBq	——
Rowe	2020	USA	P	31	63 (45–74)	0.4 (0.2–28.3)	GE Discovery RX 64 or Biograph mCT 128	^18^F-DCFPyL	<333 MBq	——
Chaussé	2020	Canada	R	93	70.4(51-87)	2.27 (0.07–51.09)	GE Discovery ST	^18^F-DCFPyL	333 ± 37 MBq	Multiple
Dietlein	2021	Germany	R	70	70.1 ± 5.5			^18^F-JK-PSMA-7	348 ± 55 MBq	——
Koschel	2021	Australia	P	98	68.0 (66.0–71.0)	0.32 (0.28–0.36)	GE Discovery 710	^18^F-DCFPyL	250 ± 50 MBq	Imaging
Perry	2021	New Zealand	R	222	71 (49–89)	0.51 (0.08–58.9)	GE Discovery 690, 710	^18^F-DCFPyL	250 ± 50 MBq	——

A multiple reference standards including biopsy, other imaging modalities and follow-up.

### Quality Assessment

**Figure 2:**
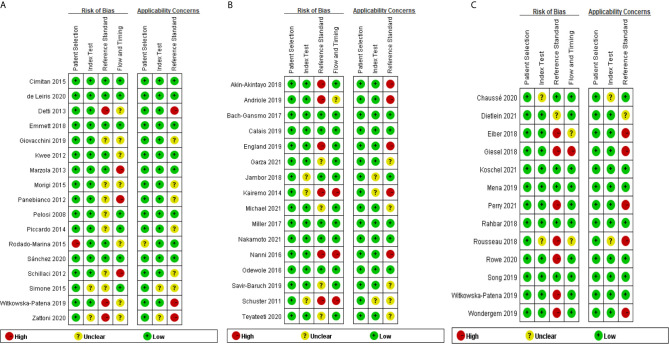
**(A)** Quality Assessment of Diagnostic Accuracy Studies-2 analysis of study bias in ^18^F-choline cohort. **(B)** Quality Assessment of Diagnostic Accuracy Studies-2 analysis of study bias in ^18^F-fluciclovine cohort. **(C)** Quality Assessment of Diagnostic Accuracy Studies-2 analysis of study bias in ^18^F prostate-specific membrane antigen (PSMA) cohort.

**Figures 2A–C** show the results of the quality assessment of each eligible study for ^18^F-choline, ^18^F-fluciclovine, and ^18^F-PSMA, respectively. Patient selection was not considered the source of bias because all studies had qualified patient selection criteria. For the index test and reference standard, some studies did not adopt the blinding method when interpreting the positive scan of the PET/CT findings, and we rated these studies as high or unclear levels regarding the risk of bias and applicability concern. Similarly, unclear or high levels were displayed on the applicability concern of flow and timing because of the different follow-up times and multiple reference standards.

### Diagnostic Performance of ^18^F-Choline and ^18^F-Fluciclovine PET/CT

**Figure 3:**
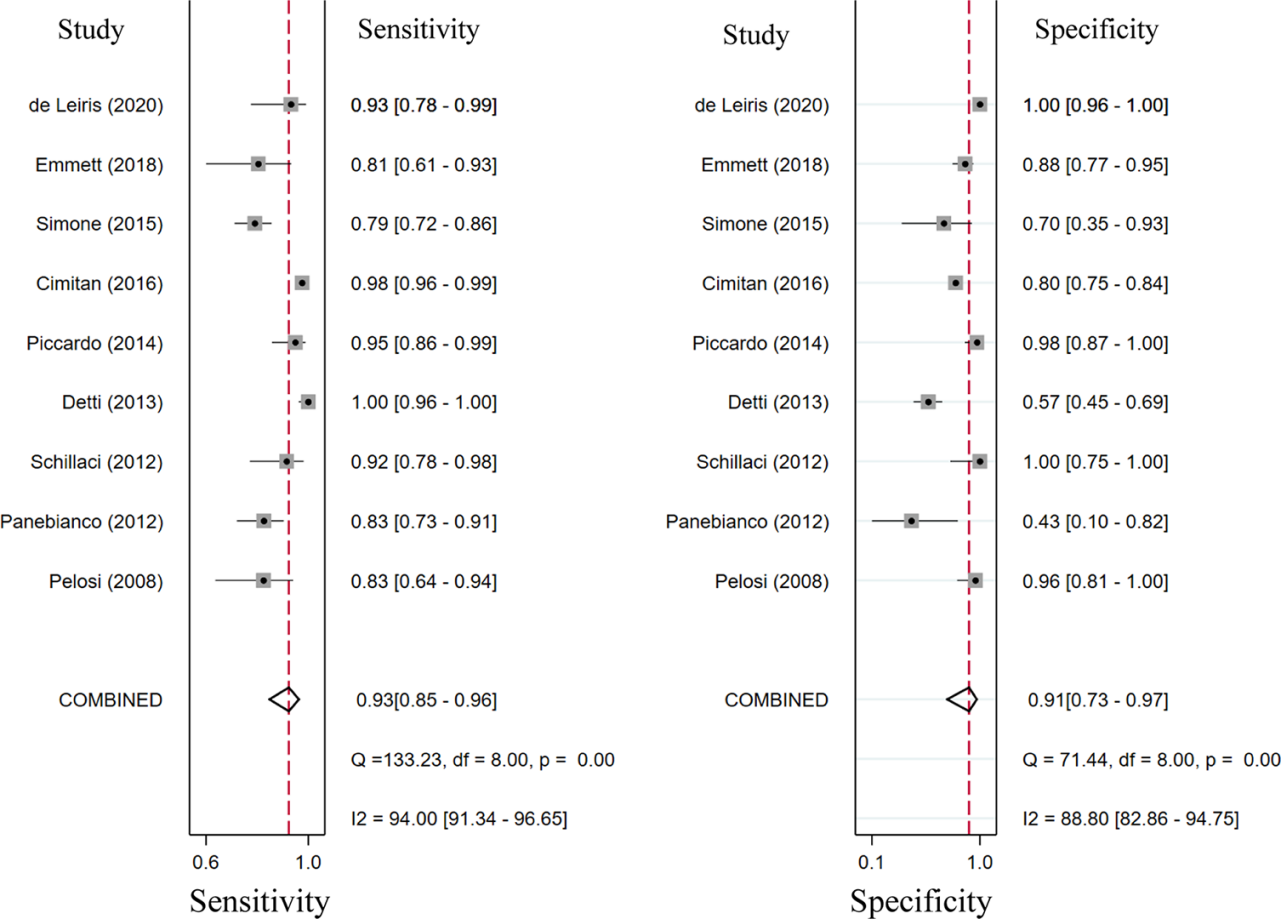
Forest plot of the proportion of ^18^F-choline positron emission tomography/computed tomography (PET/CT) sensitivity and specificity in prostate cancer patients with biochemical recurrence.

**Figure 4:**
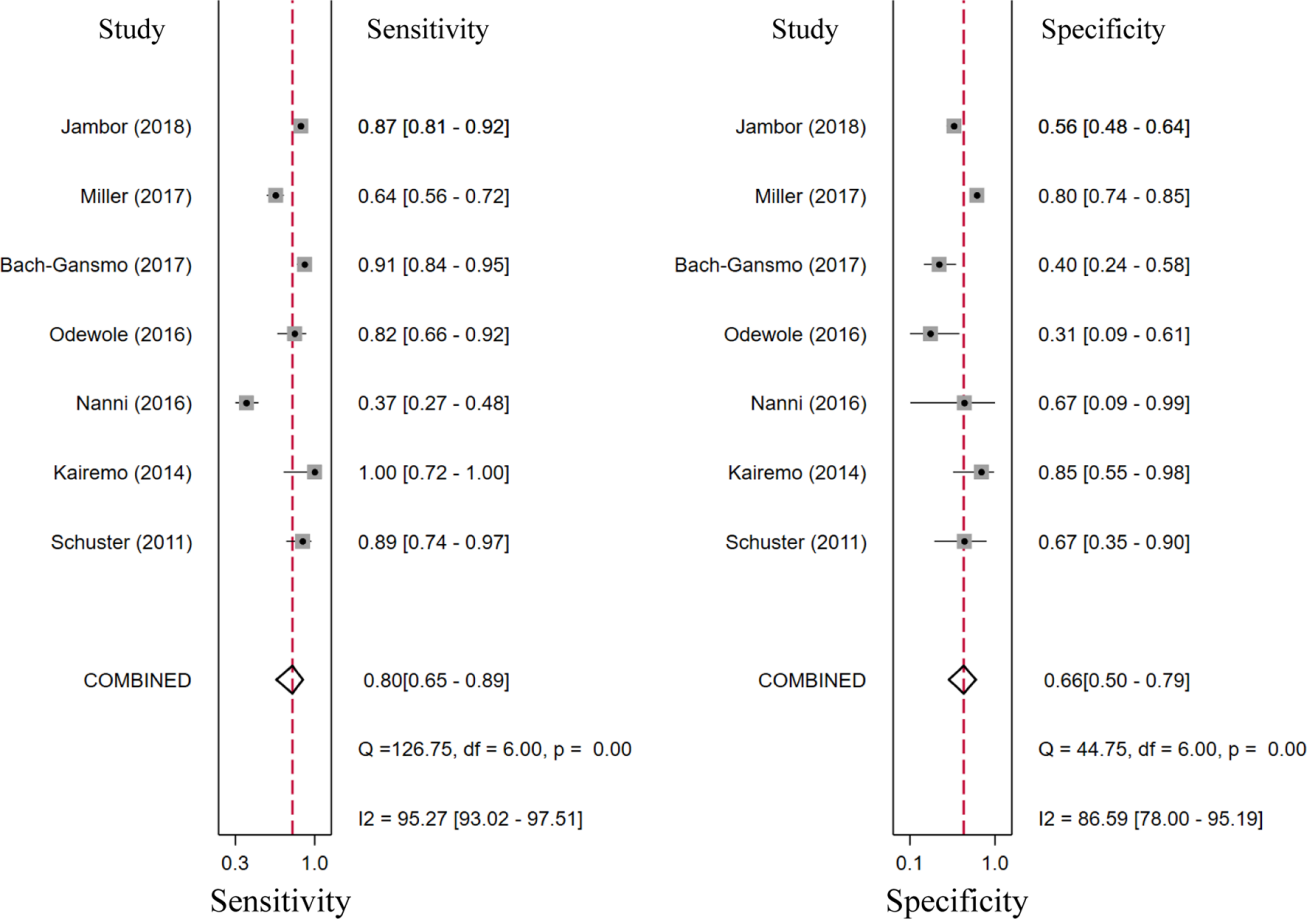
Forest plot of the proportion of ^18^F-fluciclovine PETCT sensitivity and specificity in prostate cancer patients with biochemical recurrence.

**Figure 5:**
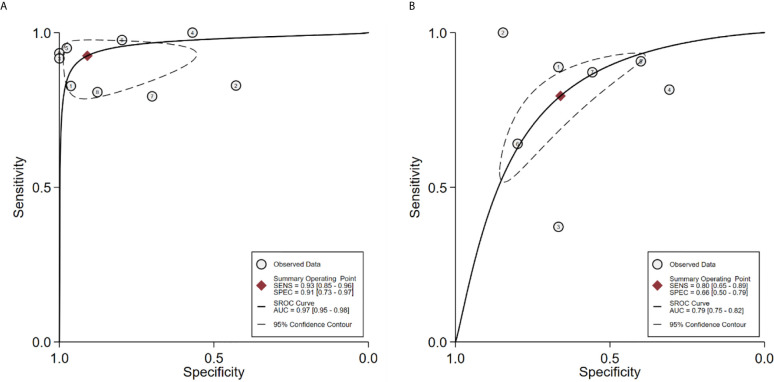
**(A)** SROC curve for the diagnostic accuracy of ^18^F-choline PET/CT in prostate cancer patients with biochemical recurrence. **(B)** SROC curve for the diagnostic accuracy of ^18^F-fluciclovine PET/CT in prostate cancer patients with biochemical recurrence. SROC, summary receiver operating characteristic.

Seventeen studies reported the diagnostic performance of ^18^F-choline, and the summary sensitivity and specificity of ^18^F-choline PET/CT in patients with BCR were 0.93 (95% CI, 0.85–0.96) and 0.91 (95% CI, 0.73–0.97), respectively (**Figure 3**). The summary sensitivity and specificity drawn from studies on ^18^F-fluciclovine were 0.80 (95% CI, 0.65–0.89) and 0.66 (95% CI, 0.50–0.79), respectively (**Figure 4**). However, the summary sensitivity and specificity were not constructed for ^18^F-PSMA PET/CT imaging because these studies mostly focused on the detection rate in patients with BCR. Summary receiver operating characteristic (SROC) curves of ^18^F-choline and ^18^F-fluciclovine were demonstrated in **Figures 5A, B**.

### Detection Rate of ^18^F-Choline, ^18^F-Fluciclovine, and ^18^F-PSMA PET/CT

**Figure 6:**
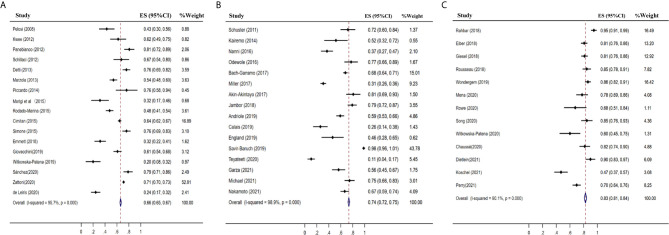
Forest plot of the proportion of ^18^F-labeled choline **(A)**, fluciclovine **(B)** and PSMA **(C)** PET/CT positivity of prostate cancer patients with biochemical recurrence.

**Figure 7:**
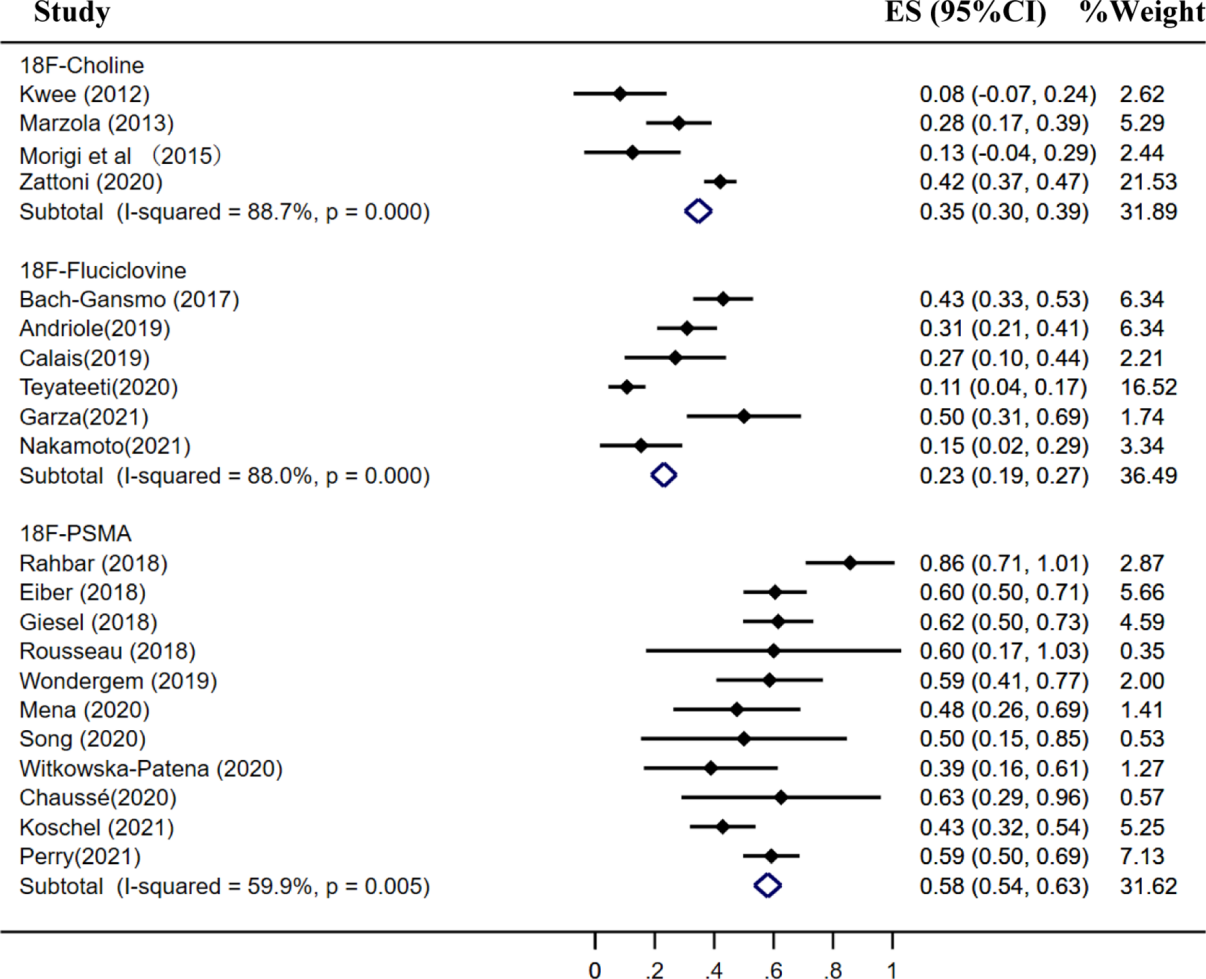
Forest plot of the proportion of ^18^F-labeled choline, fluciclovine and PSMA positivity of prostate cancer patients with BCR for PSA less than 0.5 ng/ml.

**Figure 8:**
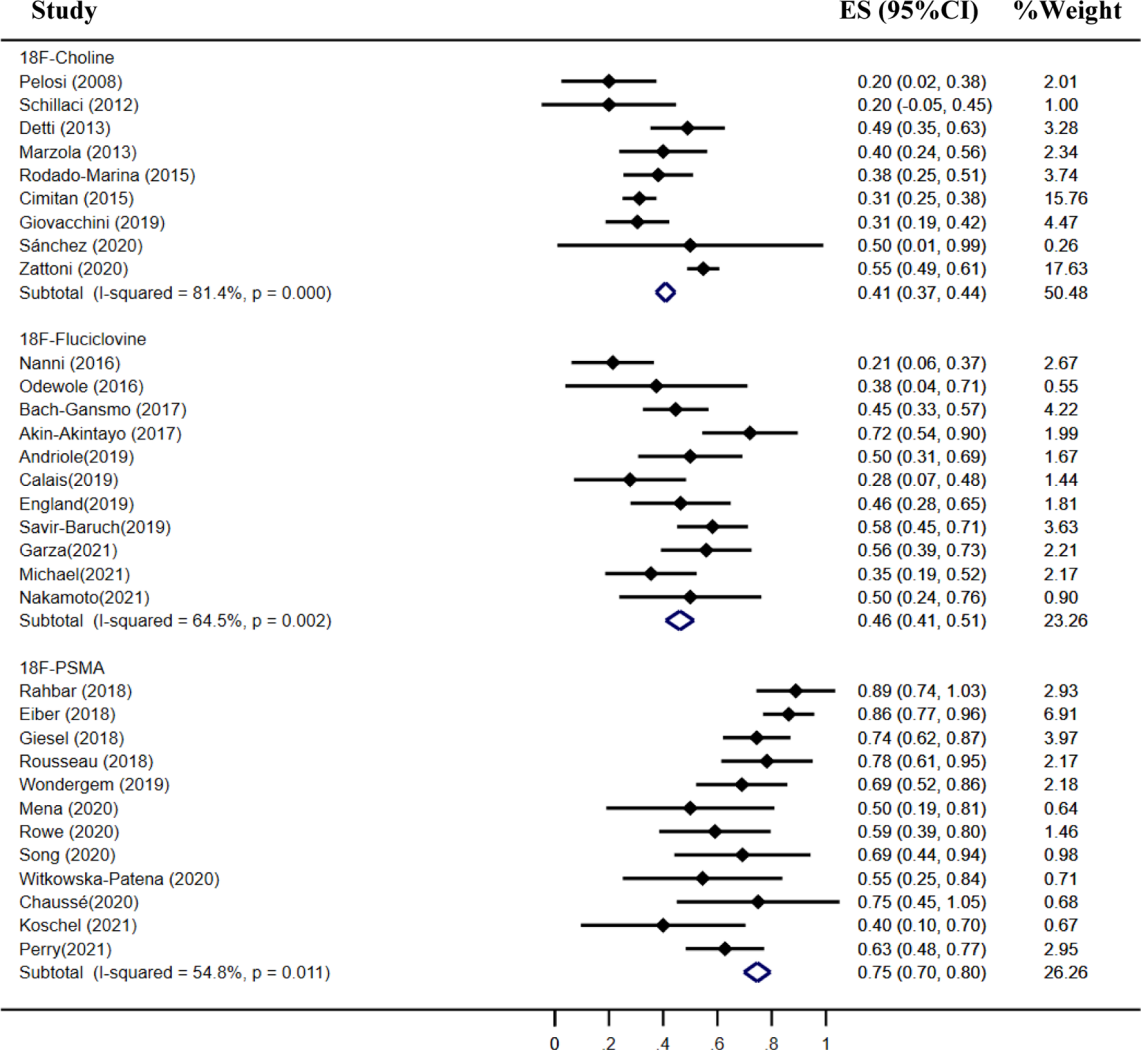
Forest plot of the proportion of ^18^F-labeled choline, fluciclovine, and PSMA positivity of prostate cancer patients with BCR for PSA 0.5–0.99 ng/ml.

**Figure 9:**
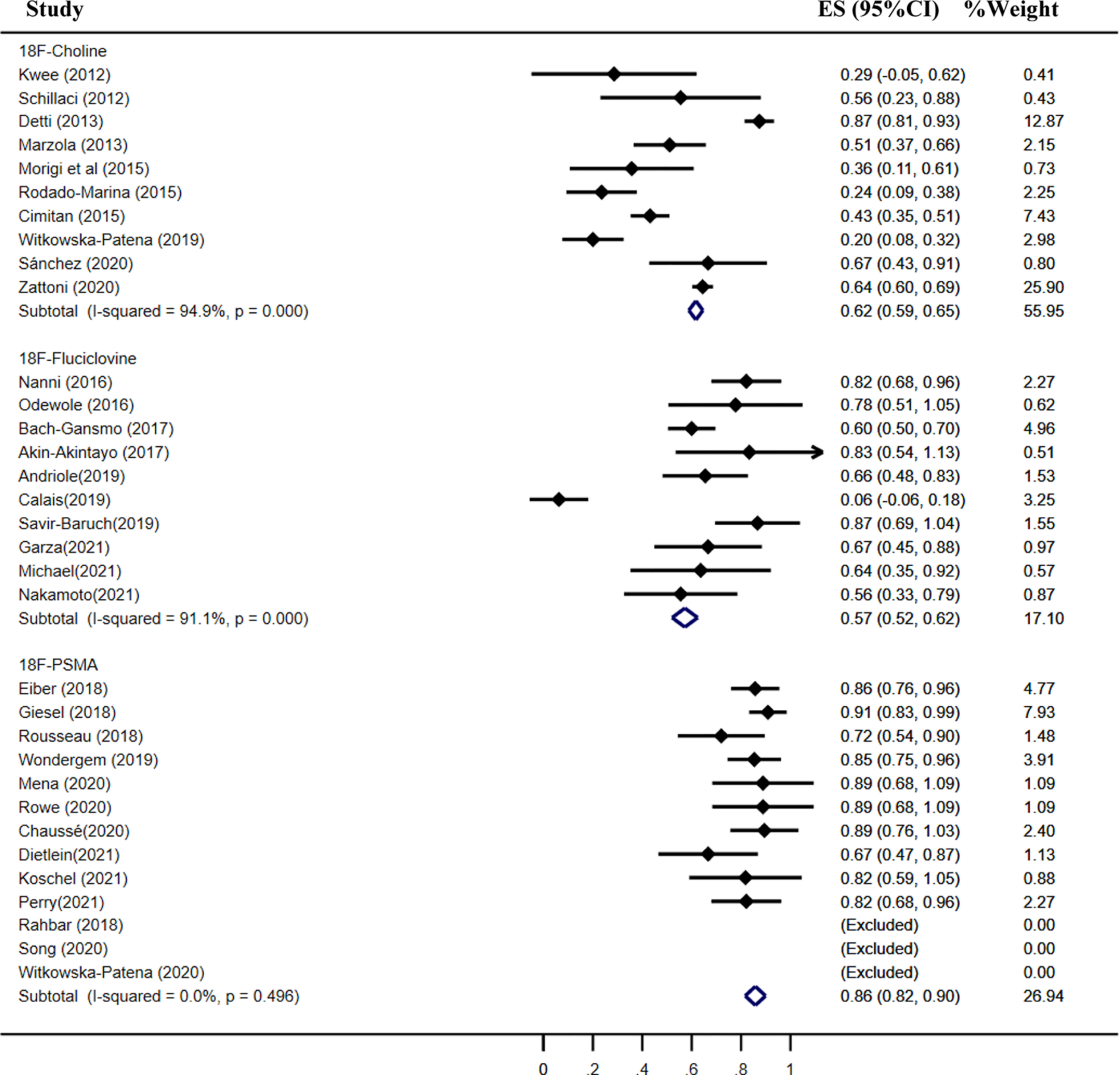
Forest plot of the proportion of ^18^F-labeled choline, fluciclovine, and PSMA positivity of prostate cancer patients with BCR for PSA 1.0–1.99 ng/ml.

**Figure 10:**
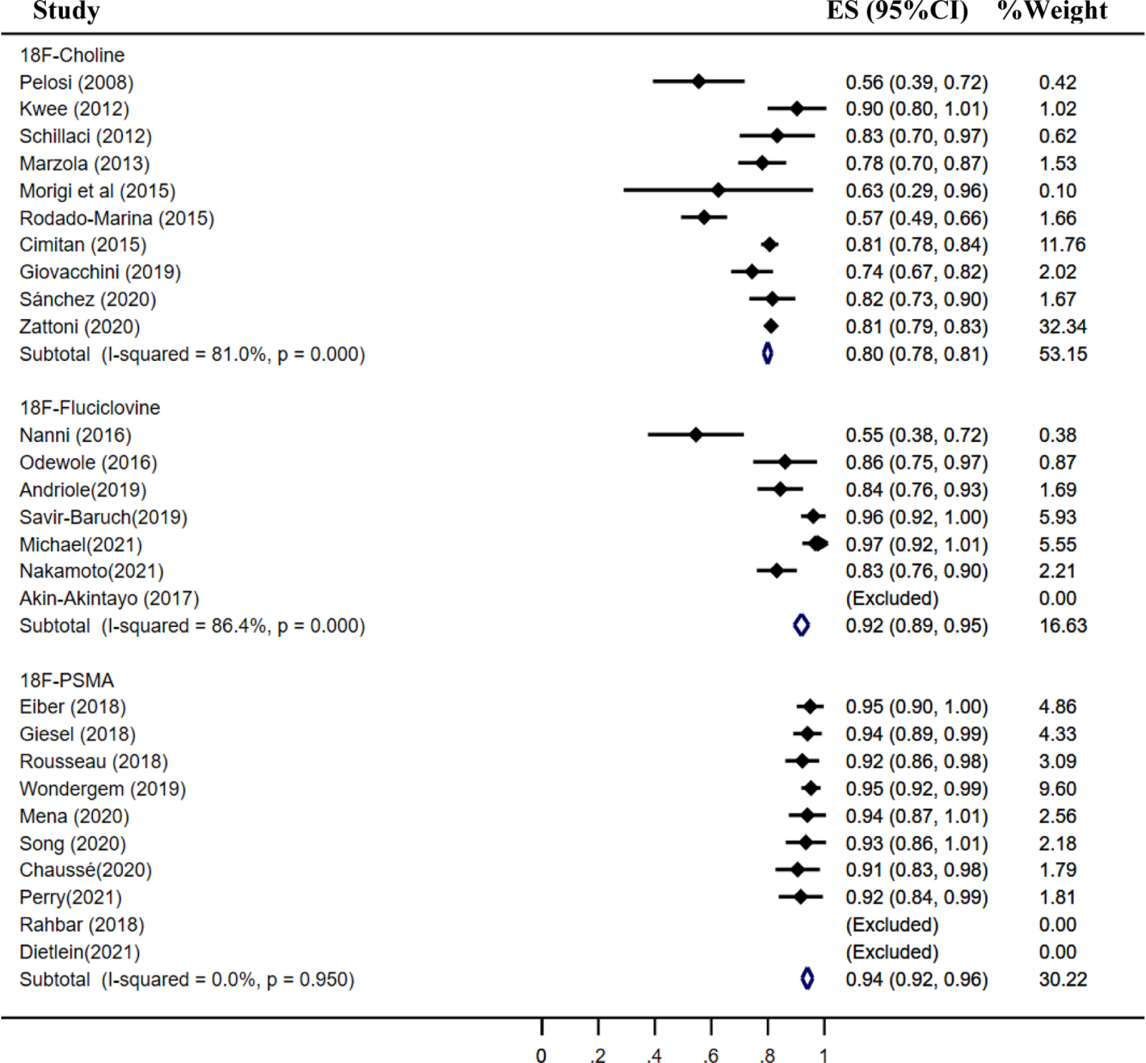
Forest plot of the proportion of ^18^F-labeled choline, fluciclovine, and PSMA positivity of prostate cancer patients with BCR for PSA more than 2.0 ng/ml.

The pooled detection rate of ^18^F-choline PET/CT was 66%, lower than 74% of ^18^F-fluciclovine PET/CT. In addition, the pooled detection rate of ^18^F-PSMA PET/CT was 83% (**Figure 6**). Meanwhile, the detection rates of ^18^F-labeled choline, fluciclovine, and PSMA were 35, 23, and 58% for a PSA level less than 0.5 ng/ml (**Figure 7**); 41, 46, and 75% for a PSA level of 0.5–0.99 ng/ml (**Figure 8**); 62, 57, and 86% for a PSA level of 1.0–1.99 ng/ml (**Figure 9**); 80, 92, and 94% for a PSA level more than 2.0 ng/ml (**Figure 10**).

## Discussion

Our meta-analysis included studies investigating the diagnostic roles of three novel ^18^F-labeled tracers applied in prostate cancer patients with BCR. From our study, the summary sensitivity and specificity of ^18^F-choline and ^18^F-fluciclovine PET/CT were 0.93 and 0.91, and 0.80 and 0.66, respectively. For the detection rate, the pooled detection rates of ^18^F-labeled choline, fluciclovine, and PSMA were 66, 74, and 83%, respectively. Meanwhile, we observed a higher detection rate of biochemically recurrent PCa with ^18^F-PSMA compared with choline and fluciclovine PET/CT for the different PSA level subgroups.

Multiple PET/CT radiotracers have been developed and experimented in recent years, motivating the wide use of PET/CT or PET/MRI in patients with PCa for staging, restaging, and response evaluation (75, 76). ^18^F-fluciclovine PET/CT showed a superior advantage over ^11^C-choline PET/CT in patients with BCR and further aid guiding decision-making in regard to patients’ treatment strategy (48, 77). In addition, PSMA PET/CT has shown superior diagnostic accuracy for recurrence and metastases of prostate cancer than fluciclovine and choline. A meta-analysis defined the diagnostic accuracy of PET/CT imaging using ^11^C-choline, ^18^F-fluciclovine, or ^68^Ga-PSMA, showing that ^68^Ga-PSMA PET/CT has a nearly equal sensitivity but the highest specificity among these tracers for PET/CT imaging in detecting biochemically recurrent PCa (25).

In contrast, our meta-analysis focused on only long-half radionuclides as ^18^F-labeled tracers and summarized the diagnostic accuracy of ^18^F-labeled choline, fluciclovine, and PSMA in detecting patients with BCR. Our study revealed that ^18^F-PSMA had the highest detection rate at different PSA levels, and the detection rate was related to the PSA level. These results were consistent with another meta-analysis that compared the detection rate of biochemically recurrent PCa between PSMA-targeted radiotracers and ^18^F-fluciclovine, finding that PSMA-targeted radiotracers demonstrate a greater detection rate than ^18^F-fluciclovine (78). A study compared prospectively paired ^18^F-fluciclovine and PSMA PET/CT scans for localizing recurrence of PCa after prostatectomy in patients with a PSA level <2.0 ng/ml (55). They found that PSMA PET/CT showed higher detection rates and should be the tracer choice when PET/CT imaging is considered for patients with biochemical recurrence after radical prostatectomy with low PSA concentrations (≤2.0 ng/ml). The same conclusion was drawn from another prospective study paired that compared ^18^F-PSMA and ^18^F-fluorocholine PET/CT in patients with BCR (42). The advantage of PSMA-targeted PET/CT imaging could be attributed to the high expression of PSMA in PCa and its metastases.

In late 2020, ^68^Ga-PSMA-11 became the first PSMA PET tracer to be approved by the FDA, which may facilitate widespread adaptation. Despite this, there also have been some limitations related to ^68^Ga-PSMA PET/CT because of the short half-life, non-ideal energies, and limited availability of ^68^Ga, limiting its clinical application in detecting occult or metastatic lesions in the prostate bed (62, 79). However, ^18^F-PSMA analogs seemed to be more favorable due to their longer half-life and a higher physical spatial resolution (23), and ^18^F-PSMA-1007, as a second-generation ^18^F-labeled PSMA tracer, demonstrated high labeling yields, better tumor uptake, and hepatobiliary excretion, making it an ideal PSMA-target tracer for diagnostic imaging in patients with BCR (21, 23). Our meta-analysis found the pooled detection rate with ^18^F-PSMA of 58% for a PSA level of less than 0.5 ng/ml, 75% for a PSA level of 0.5 to 0.99 ng/ml, and 86% for a PSA level of 1.0 to 1.99 ng/ml. These detection rates are equal or higher than those in recent studies involving ^68^Ga PSMA PET/CT (80, 81).

Compared with FDA approval of ^68^Ga-PSMA-11 in late 2020, ^11^C-choline and ^18^F-fluciclovine PET/CT have one temporary advantage as they have been granted FDA approval early. They were more accessible and used in the US and Europe. Many studies compared the diagnostic utility of ^18^F-fluciclovine with ^11^C-choline PET/CT imaging, showing a better performance in terms of lesion detection rate (48, 82). A recent meta-analysis demonstrated that ^18^F-fluciclovine had the similar sensitivity and detection rate compared with ^11^C-choline, but lower specificity than ^11^C-choline (83). Unlike the short physical half-life of ^11^C-choline, the radiofluorine of ^18^F-choline provides a long physical half-life (109.8 min), allowing for centralized manufacture and distribution. These intrinsic advantages of ^18^F labeling has made ^18^F-choline PET/CT valuable in staging patients with PCa and detecting recurrently PCa metastases after initial treatment (33, 84). There were limited studies in comparing directly to determine which imaging modality has a better diagnostic efficiency between ^18^F-choline and ^18^F-fluciclovine. In our meta-analysis, ^18^F-choline had a higher sensitivity and specificity than ^18^F-fluciclovine through assessing the summary sensitivity and specificity. ^18^F-choline also has better detection rates than ^18^F-fluciclovine at PSA levels under 0.5 ng/ml and 1.0–1.99 ng/ml, but the pooled detection rate of ^18^F-fluciclovine was higher than that of ^18^F-choline in biochemically recurrent PCa. This difference could be interpreted by different biological processes between amino acid transport and choline expression.

## Limitations

There were several limitations to this study that should be mentioned. First, we only evaluated the diagnostic accuracy of both ^18^F-choline and ^18^F-fluciclovine PET/CT in patients with BCR, and the pooled sensitivity and specificity for ^18^F-PSMA PET/CT were not feasible because of insufficient published data. Second, there were significant heterogeneities among institutions, PET/CT scanners, radiotracers, and prior treatment of patients, which increased the risk of bias and led to significant heterogeneity among ^18^F-choline, ^18^F-fluciclovine and ^18^F-PSMA PET/CT. Third, most of the included studies were retrospective analyses, had small sample sizes, had limited reference standards, and lacked prospective, large sample, and interagent comparison studies. Fourth, there was publication bias according to Egger’s test regarding the included studies of ^18^F-choline, ^18^F-flocilovine, and ^18^F-PSMA PET/CT, limiting the interpretation of the data to some degree.

## Conclusion

PET/CT imaging with ^18^F-choline, ^18^F-fluciclovine, and ^18^F-PSMA is promising in detecting prostate cancer patients with BCR. ^18^F-PSMA PET/CT demonstrated a significantly higher detection rate over ^18^F-choline and ^18^F-fluciclovine for different PSA levels, particularly in PSA level less than 2.0 ng/ml.

## Data Availability Statement

The original contributions presented in the study are included in the article/supplementary material. Further inquiries can be directed to the corresponding author.

## Author Contributions

Conceived and designed the experiments: RW, GS, and RT. Performed the experiments: GS, RW, and MH. Analyzed the data: RW, GS, and RT. Analyzed tools: GS and MH. Wrote the paper: RW and RT. Provided critical input into the design and drafting of the manuscript: RW, GS, and RT. All authors contributed to the article and approved the submitted version.

## Funding

This study was supported by the National Natural Science Foundation of China (81971653).

## Conflict of Interest

The authors declare that the research was conducted in the absence of any commercial or financial relationships that could be construed as a potential conflict of interest.
